# CNN-based framework for Alzheimer's disease detection from EEG via dynamic mode decomposition

**DOI:** 10.3389/fninf.2025.1706099

**Published:** 2025-11-20

**Authors:** Jacob Kang, Hunseok Kang, Jong-Hyeon Seo

**Affiliations:** 1 College of Computer, Mathematical, and Natural Sciences, University of Maryland, College Park, MD, United States; 2 College of Engineering and Technology, American University of the Middle East, Egaila, Kuwait; 3 School of Basic Sciences, Hanbat National University, Daejeon, Republic of Korea

**Keywords:** convolution neural network (CNN), electroencephalography (EEG), brain dynamics, fast Fourier transformation (FFT), Alzheimer's disease (AD), cognitive disorders, Dynamic Mode Decomposition (DMD), open-eyes EEG

## Abstract

Alzheimer's disease (AD) and frontotemporal dementia (FTD) are major neurodegenerative disorders with characteristic EEG alterations. While most prior studies have focused on eyes-closed (EC) EEG, where stable alpha rhythms support relatively high classification performance, eyes-open (EO) EEG has proven particularly challenging for AD, as low-frequency instability obscures the typical spectral alterations. In contrast, FTD often remains more discriminable under EO conditions, reflecting distinct neurophysiological dynamics between the two disorders. To address this challenge, we propose a CNN-based framework that applies Dynamic Mode Decomposition (DMD) to segment EO EEG into shorter temporal windows and employs a 3D CNN to capture spatio-temporal-spectral representations. This approach outperformed not only the conventional short-epoch spectral ML pipeline but also the same CNN architecture trained on FFT-based features, with particularly pronounced improvements observed in AD classification. Excluding delta yielded small gains in AD-involving contrasts, whereas FTD/CN was unchanged or slightly better with delta retained—suggesting delta is more perturbative in AD under EO conditions.

## 1 Introduction

Alzheimer's disease (AD) and frontotemporal dementia (FTD) are two of the most common progressive neurodegenerative disorders, predominantly affecting older adults and leading to memory loss, cognitive decline, and behavioral impairments (Levy et al., 1996; Ntetska et al., 2025; Perry and Hodges, 2000; Zuin et al., 2024). While AD is primarily characterized by memory deterioration, language deficits, and visuospatial dysfunction, FTD manifests early through behavioral changes such as disinhibition, apathy, compulsivity, and language impairment, with relative preservation of memory in the early course (Perry and Hodges, 2000; Nishida et al., 2011). Despite differences, the two disorders exhibit overlapping symptoms, complicating diagnosis. Currently, there are no curative treatments for either condition, making early and accurate diagnosis of paramount importance.

Diagnosis of AD and FTD typically involves neuropsychological testing, magnetic resonance imaging (MRI), and fluorodeoxyglucose positron emission tomography (FDG-PET). Although effective, these methods are costly, not universally accessible, and limited in their sensitivity during the early stages (Davatzikos et al., 2008; Jack et al., 2018; Jiang, 2023). Electroencephalography (EEG), on the other hand, is a low-cost, non-invasive, and widely accessible tool that captures neural activity with millisecond-level temporal resolution. Characteristic EEG changes, such as posterior alpha rhythm slowing and increased theta/delta power, have been consistently reported in AD and FTD (Jeong, 2004; Musaeus et al., 2018; Nishida et al., 2011).

In parallel, recent advances in machine learning (ML) and deep learning (DL) have enabled automatic classification of EEG signals, uncovering spatial and temporal patterns indicative of neurodegenerative disorders (AlSharabi et al., 2023; Cansiz et al., 2025; Khan et al., 2025; Zhang et al., 2025). Studies have reported abnormalities in oscillatory dynamics and functional connectivity in AD and FTD patients using ML/DL models (Adebisi et al., 2023; Afshari and Jalili, 2017).

In particular, a publicly available dataset of resting-state EEG recordings under eyes-closed (EC) conditions (Miltiadous et al., 2023) and its extension with CNN-based classification (Stefanou et al., 2025) have demonstrated the potential of EEG-driven computational models for dementia detection.

According to Stefanou et al. (2025), the authors proposed a novel CNN-based framework for Alzheimer's disease detection that employed EEG spectrogram representations under eyes-closed (EC) conditions. Specifically, they transformed EEG recordings into time–frequency spectrograms (using FFT) and used these as inputs to a convolutional neural network. Their approach achieved robust classification performance in distinguishing AD, FTD, and healthy controls (CN), validated with a leave-N-subjects-out (LNSO) scheme—79.45% for AD/CN, 72.85% for FTD/CN, and 80.69% for AD+FTD/CN—underscoring the utility of eyes-closed spectrogram-based CNNs for dementia EEG.

Most EEG-based biomarkers of dementia have historically been derived from eyes-closed (EC) recordings, where stable alpha rhythms provide reliable spectral features (Babiloni et al., 2017; Rossini et al., 2020). In contrast, eyes-open (EO) EEG has been far less studied due to reduced alpha activity, greater variability, and susceptibility to ocular and attentional artifacts, making it more challenging to analyze (Ntetska et al., 2025). The recent release of an EO EEG dataset under photostimulation by Ntetska et al. (2025) provides a timely opportunity to explore this condition, which captures neural dynamics distinct from EC.

Whereas EC recordings are typically dominated by posterior alpha rhythms reflecting a relaxed resting state, EO EEG exhibits marked alpha suppression alongside increased theta and beta activity, reflecting attentional and cognitive engagement. Importantly, Ntetska et al. reported that AD and FTD patients showed reduced alpha suppression compared to controls, indicating impaired neural reactivity to visual stimulation. These findings highlight that EO EEG provides distinct and clinically relevant neural dynamics, underscoring the importance of developing tailored analytic approaches.

However, conventional FFT-based spectrograms, while effective in EC conditions, tend to overemphasize low-frequency power in EO recordings, potentially obscuring other relevant patterns. To address this limitation, we propose a CNN-based framework that incorporates novel features derived from Dynamic Mode Decomposition (DMD). Unlike FFT, DMD captures spatio-temporal coherent modes and thus provides a representation that emphasizes dynamic neural characteristics beyond static frequency-domain power. This approach is expected to yield complementary biomarkers for dementia classification by better characterizing the unique dynamics of EO EEG.

In previous CNN studies on the EC dataset, spectrograms derived from longer windows (e.g., 30-s epochs) outperformed shorter windows (Stefanou et al., 2025), consistent with findings that longer epochs improve spectral reliability (Ng et al., 2022). By contrast, our EO recordings contain sequential photic stimulation, so long windows would mix multiple stimulus conditions and blur nonstationary dynamics: SSVEP(Steady-State Visually Evoked Potential) responses exhibit time-ordered, frequency-dependent changes in phase synchrony and propagation (Norcia et al., 2015; Tsoneva et al., 2021). Accordingly, we do not model long series directly. Instead, we deliberately avoid long-window aggregation and apply Dynamic Mode Decomposition (DMD) to short, *non-overlapping* 2-s slices, obtaining coherent spatiotemporal modes with identifiable oscillation frequencies beyond per-channel power (Schmid, 2010; Tu et al., 2014). The slice-wise DMD mode-magnitude maps are then stacked in order to form a 3D tensor for the CNN, preserving segment-to-segment evolution while preventing stimulus mixing and reducing the computational burden of long-horizon DMD.

Because SSVEP responses are sensitive to stimulus paradigm and frequency (Norcia et al., 2015), we treat *subject-specific stimulus heterogeneity* as a nuisance and adopt a stimulus-agnostic, uniform epoching strategy. Within a single slice-based pipeline, recordings are partitioned uniformly within stimulus-on periods into non-overlapping short slices for feature extraction, so that stimulus composition is not explicitly stratified or encoded as a predictive cue. For a like-for-like comparison, we also derive an FFT-based spectrotemporal representation using the same epoching and slicing scheme in place of DMD; aside from the feature extractor (DMD vs. FFT), the classifier configuration, input shape, and training protocol are identical, and matched-dimensionality tensors are fed to the same CNN. Consistent with this design choice, we verified on the dataset—via class–category distribution analysis—that stimulus composition did not materially bias between-group comparisons.

While DMD provides a representation that captures spatio-temporal modes beyond conventional frequency power, a further consideration is the role of low-frequency activity in EO EEG. In previous EC-based CNN studies (Stefanou et al., 2025), spectrogram features yielded high classification accuracy; however, when the same methodology was applied to the EO dataset (Ntetska et al., 2025), the performance for AD was substantially lower. We attribute this reduction to the disproportionate influence of delta activity (0.5–4 Hz, as defined in Ntetska et al., 2025) in EO recordings, which may obscure disease-relevant dynamics.

To test this hypothesis, we conducted controlled comparisons using both FFT- and DMD-based representations, evaluating conditions with and without the delta-band (0.5–40 Hz vs. 4–40 Hz). By applying the same CNN framework across all feature sets, we were able to directly assess the extent to which excluding delta activity improves classification performance in EO EEG.

A critical issue in EEG-based deep learning for dementia classification lies in the choice of evaluation methodology. Several earlier studies employed segment-based or random cross-validation procedures, in which EEG segments from the same subject could appear in both the training and test sets, leading to data leakage and overly optimistic performance estimates (Brookshire et al., 2024). Brookshire et al. explicitly demonstrated that such leakage can dramatically inflate classification accuracy in Alzheimer's studies, and strongly recommended subject-wise validation strategies to avoid identity confounding. Following this recommendation, the EC-based CNN spectrogram study adopted a subject-wise scheme, namely (LNSO), where entire subjects are excluded from the training set whenever they are used for testing (Stefanou et al., 2025).

The importance of subject-wise partitioning has been widely emphasized in the EEG literature. For example, Zanola et al. (2025) showed that nested leave-*N*-subjects-out (LNSO) validation provides more reliable performance estimates than non-nested approaches that are prone to overfitting. Similarly, Kunjan et al. (2021) demonstrated that subject-level cross-validation (e.g., LOSO) yields substantially more robust generalization estimates than random k-fold validation in EEG-based disease diagnosis. Building on this evidence, we adopted an LNSO validation framework to ensure fair and reliable evaluation across participants, thereby avoiding inflated metrics and enhancing the credibility of the reported results.

Therefore, in this study, we introduce a CNN-based framework that incorporates DMD-derived features for analyzing EO EEG in dementia classification. By addressing the limitations of FFT-based representations and ensuring rigorous subject-wise evaluation, our work contributes a novel perspective on the role of EO EEG as a complementary biomarker for AD and FTD.

## 2 Materials and methods

This section provides a detailed description of the dataset, feature construction process, and classification framework adopted in this study. We first introduce the dataset of stimulus-related EEG recordings, including its characteristics and the criteria used for subject inclusion and exclusion. Next, we describe the Dynamic Mode Decomposition (DMD) procedure applied to the segmented EEG data, which transforms each epoch into a set of spatio-temporal modes. The subsequent feature extraction step outlines how DMD-derived representations were converted into fixed-size images, together with the construction of alternative FFT-based features for comparative analysis. We then present the CNN model architecture used for classification, along with the baseline algorithms against which our approach was evaluated. Finally, we detail the validation methodology, emphasizing the use of subject-wise partitions to ensure a fair and reliable assessment of classification performance.

### 2.1 Dataset

This study used scalp EEG recordings from a publicly available dataset (OpenNeuro, dataset ID: ds006036, version 1.0.4; DOI: 10.18112/openneuro.ds006036.v1.0.4, which was updated in April 2025. EEG signals were collected using 19 Ag/AgCl electrodes (Fp1, Fp2, F7, F3, Fz, F4, F8, T3, C3, Cz, C4, T4, T5, P3, Pz, P4, T6, O1, and O2) by the international 10–20 system and sampled at 500 Hz with a resolution of 10 μV/mm. A total of 88 participants were included in this study, comprising 36 patients with Alzheimer's disease (AD), 23 patients with frontotemporal dementia (FTD), and 29 healthy controls (CN).

The international Mini-Mental State Examination (MMSE) was used to evaluate the cognitive and neuropsychological status of subjects, with scores ranging from 0 to 30 (lower scores indicating more severe impairment). The AD group (12 males, 24 females) had a mean age of 66.4 years (SD = 7.9) and an average MMSE score of 17.75 (SD = 4.5). The FTD group (14 males, 9 females) had a mean age of 63.7 years (SD = 8.2) with an average MMSE score of 22.17 (SD = 2.6). The CN group (18 males, 11 females) had a mean age of 67.9 years (SD = 5.4), all scoring 30 on the MMSE. Data acquisition was ethically approved by the Scientific and Ethical Committee of the Aristotle University of Thessaloniki and AHEPA University Hospital (protocol number: 142/12-04-2023).

*Note that* in our experiments four recordings with insufficient usable duration ( ≤ 30 s; IDs 15, 64, 65, 78) were excluded; accordingly, all analyses use the post-exclusion sample with the following demographics: AD (11M/24F), age 66.5 ± 8.0 y, MMSE 17.7 ± 4.6; FTD (13M/9F), age 63.7 ± 8.4 y, MMSE 22.2 ± 2.7; CN (17M/10F), age 67.9 ± 5.6 y, MMSE 30.

While this dataset provides both raw and preprocessed EEG signals, many previous studies have utilized the preprocessed version, which includes noise filtering and artifact removal (see Ntetska et al., 2025). In this study, we also employed the preprocessed EEG signals. Consequently, no additional preprocessing steps were applied during the feature extraction stage, as the provided data were already cleaned and ready for analysis.

Furthermore, since our focus was on evaluating brain responses to visual stimulation, only EEG segments corresponding to visual stimulus events were selected for analysis. Specifically, for each subject, we identified periods during which visual stimuli were presented and extracted EEG data exclusively within these intervals. The detailed onset/offset boundaries for each subject and stimulus frequency are reported in the Photic stimulation intervals by frequencies table (Appendix Table 5). As shown in Table 1, the resulting segments do not have a uniform duration across subjects. Consequently, some recordings with relatively short durations could not be included in subsequent analyses, as our methodology required sufficiently long segments to ensure reliable evaluation of stimulus-related brain dynamics.

**Table 1:** Summary of visual stimulus onset and offset times (in seconds) and the corresponding stimulus durations for each subject in the Alzheimer's disease (AD), Normal control (CN), and Frontotemporal dementia (FTD) groups.

**Alzheimer's disease (AD)**			**Normal control (CN)**			**Frontotemporal dementia (FTD)**		
**ID**	**[Onset, Offset]**	**Duration**	**ID**	**[Onset, Offset]**	**Duration**	**ID**	**[Onset, Offset]**	**Duration**
1	[3.80, 73.79]	69.99	37	[6.48, 76.47]	69.99	66	[1.26, 91.25]	89.99
2	[17.39, 107.38]	89.99	38	[14.82, 84.82]	69.99	67	[16.01, 106.00]	89.99
3	[0.03, 45.49]	45.46	39	[14.94, 165.01]	150.07	68	[0.30, 90.29]	89.99
4	[15.35, 105.34]	89.99	40	[0.52, 70.51]	69.99	69	[14.25, 104.24]	89.99
5	[5.37, 94.87]	89.50	41	[19.09, 109.08]	89.99	70	[7.11, 97.10]	89.99
6	[14.37, 123.38]	109.01	42	[16.49, 106.49]	89.99	71	[3.88, 93.88]	89.99
7	[17.39, 107.39]	89.99	43	[7.25, 157.78]	150.53	72	[0.95, 90.94]	89.99
8	[15.57, 105.56]	89.99	44	[9.52, 99.52]	89.99	73	[18.57, 108.56]	89.99
9	[21.89, 111.88]	89.99	45	[1.51, 126.46]	124.96	74	[4.23, 94.22]	89.99
10	[15.08, 105.07]	89.99	46	[14.29, 104.28]	89.99	75	[12.63, 122.50]	109.86
11	[13.99, 103.98]	89.99	47	[14.33, 104.33]	89.99	76	[10.85, 100.67]	89.81
12	[2.99, 92.98]	89.99	48	[4.57, 94.56]	89.99	77	[8.49, 98.48]	89.99
13	[17.18, 107.17]	89.99	49	[27.09, 117.08]	89.99	78	[8.11, 38.10]	30.00
14	[10.05, 100.04]	89.99	50	[9.49, 99.48]	89.99	79	[14.65, 84.64]	69.99
15	[0.45, 30.45]	30.00	51	[12.25, 102.24]	89.99	80	[8.56, 78.55]	69.99
16	[5.05, 95.04]	89.99	52	[2.08, 92.07]	89.99	81	[21.90, 91.89]	69.99
17	[19.54, 109.53]	89.99	53	[10.55, 100.54]	89.99	82	[3.81, 73.80]	69.99
18	[24.73, 114.72]	89.99	54	[3.83, 153.64]	149.81	83	[1.40, 71.39]	69.99
19	[0.68, 90.67]	89.99	55	[15.39, 105.38]	89.99	84	[2.29, 72.28]	69.99
20	[0.95, 90.95]	89.99	56	[17.39, 107.38]	89.99	85	[29.51, 99.50]	69.99
21	[18.10, 108.09]	89.99	57	[0.06, 69.45]	69.39	86	[9.77, 79.76]	69.99
22	[4.05, 94.04]	89.99	58	[0.05, 61.44]	61.39	87	[5.51, 75.50]	69.99
23	[2.92, 72.91]	69.99	59	[0.45, 70.44]	69.99	88	[58.40, 128.39]	69.99
24	[7.33, 77.32]	69.99	60	[0.74, 130.71]	129.97			
25	[3.81, 73.80]	69.99	61	[6.05, 76.04]	69.99			
26	[12.33, 82.32]	69.99	62	[16.58, 86.57]	69.99			
27	[4.97, 94.96]	89.99	63	[2.74, 72.73]	69.99			
28	[3.89, 73.88]	69.99	64	[0.03, 23.48]	23.45			
29	[34.24, 104.23]	69.99	65	[0.03, 20.28]	20.25			
30	[1.59, 71.58]	69.99						
31	[7.27, 77.26]	69.99						
32	[2.79, 72.78]	69.99						
33	[25.86, 95.85]	69.99						
34	[16.08, 86.07]	69.99						
35	[18.33, 108.32]	89.99						
36	[5.32, 75.32]	69.99						

*Note that* as shown in Table 1, usable segment durations vary across subjects. To obtain stable subject-level estimates under LNSO, we required that each recording allow construction of at least one ≥24 s epoch after preprocessing; recordings with total usable duration < 30 s were excluded. This threshold was motivated by the empirical duration distribution and a brief sensitivity check, which indicated that including very short recordings led to unstable estimates and a noticeable drop in accuracy. Applying this rule excluded four short recordings (Subject IDs: 15, 64, 65, and 78) from subsequent analyses.

### 2.2 Dynamic mode decomposition

One established use of Dynamic Mode Decomposition (DMD) is to characterize the temporal evolution of high-dimensional signals (Schmid, 2010; Tu et al., 2014), with EEG/ERP applications demonstrating phase-consistent dynamics captured by DMD (Li et al., 2022). DMD decomposes the changing patterns of the signal into fundamental elements known as “dynamic modes.” These modes represent the characteristics of the signal as it varies over time, and each mode represents the movement of a specific frequency within the signal (Schmid, 2010).

The EEG signal is decomposed into a sum of signals in the DMD mode using DMD, and the filtering is performed by reconstructing the signal with only the modes that meet the filtering parameters. We briefly explain the DMD and its components, which are the results needed for decomposing the EEG signal. In addition, we specify the eigenfrequency of the decomposed mode signals to be used for filtering.

#### 2.2.1 Mathematical formulation

Dynamic Mode Decomposition (DMD) is a technique used to slice states distinguished by dynamic modes. These modes consist of empirically derived vectors, extracted directly from the data, a process elaborated in Tu et al. (2014). Fundamentally, DMD operates as a method for order reduction, proficient in distilling the intrinsic dynamics present in multidimensional complex systems by isolating specific frequencies, as explored in Dang et al. (2018).

Consider a time series


$$\begin{array}{l} X:={\left\{\text{x}\left(t_{k}\right)\right\}}_{k=1}^{N}, \end{array}$$ (1)

where **x**(*t*_*k*_) belongs to ℝ^*M*^, and the time interval between sample points *t*_*k*+1_−*t*_*k*_ is fixed at Δ*t*. For a given signal *X* in Equation 1, the (*MS*) × (*N*_*S*_) shift-stack Hankel matrix **Y**^(*S*)^ is constructed as:


$$\begin{array}{l} {\text{Y}}^{\left(s\right)}:=\left[\begin{array}{cccc} \text{x}\left(t_{1}\right) & \text{x}\left(t_{2}\right) & \cdots & \text{x}\left(t_{N_{S}}\right)\\ \text{x}\left(t_{2}\right) & \text{x}\left(t_{3}\right) & \cdots & \text{x}\left(t_{N_{S}+1}\right)\\ \vdots & \vdots & \ddots & \vdots \\ \text{x}\left(t_{S}\right) & \text{x}\left(t_{S+1}\right) & \cdots & \text{x}\left(t_{N}\right) \end{array}\right]=\left[{\text{y}}_{1}\;{\text{y}}_{2}\;\cdots \;{\text{y}}_{N_{S}}\right] \end{array}$$ (2)

where *N*_*S*_: = *N*−*S*+1 and *S* denotes the predetermined stack size. To encapsulate the maximal spectrum and temporal complexity of the original signal, it is imperative to maximize the dimensions of (*MS*) × (*N*_*S*_).

The DMD algorithm accomplishes a low-rank eigendecomposition of the matrix **A** by optimally approximating **y**_*k*_ in the least squares sense, minimizing the following:


$$\begin{array}{l} ||{\text{y}}_{k+1}-\text{A}{\text{y}}_{k}||. \end{array}$$ (3)

To diminish Equation 3, the *N*_*S*_ column vectors are assembled into two data matrices with size (*MS*) × (*N*_*S*_−1):


$$\begin{array}{l} {\text{Y}}_{1}=\left[{\text{y}}_{1}\;{\text{y}}_{2}\;\cdots \;{\text{y}}_{N_{S}-1}\right],\;{\text{Y}}_{2}=\left[{\text{y}}_{2}\;{\text{y}}_{3}\;\cdots \;{\text{y}}_{N_{S}}\right] \end{array}$$

Subsequently, the local linear approximation can be articulated as:


$$\begin{array}{l} {\text{Y}}_{2}\approx \text{A}{\text{Y}}_{1}, \end{array}$$ (4)

The resolution to Equation 4 entails discovering **A** that minimizes:


$$\begin{array}{l} ||{\text{Y}}_{2}-\text{A}{\text{Y}}_{1}{||}_{\text{Frobenius}}. \end{array}$$

#### 2.2.2 Eigen decomposition and mode calculation

Rather than conducting the eigendecomposition of **A** directly, the DMD algorithm employs a low-dimensional surrogate, $\tilde{\text{A}}$, via Singular Value Decomposition (SVD) (Dang et al., 2018; Faires and Burden, 2012) of **Y**_1_:


$$\begin{array}{l} \tilde{\text{A}}=\text{ΦΛ}{\text{Φ}}^{-1} \end{array}$$ (5)

where $\text{Λ}=\text{diag}\left(\lambda_{1},\lambda_{2},\ldots ,\lambda_{R}\right)\in {\mathbb{C}}^{R\times R}$ is a diagonal matrix containing *R*(≤ *N*_*S*_) eigenvalues of **A**, and **Φ**∈ℂ^(*MS*) × (*R*)^ denotes the DMD modes.

In Equation 5, each snapshot, **y**_*k*+1_ can be approximated as:


$$\begin{array}{l} {\text{y}}_{k+1}\approx \tilde{\text{A}}{\text{y}}_{k} \end{array}$$

for *k* = 1, 2, …, *N*_*S*_−1. Hence, the matrix $\tilde{\text{A}}$ furnishes an approximation of the sample data, decomposing it into a unified space-time matrix:


$$\begin{array}{l} {\text{y}}_{k}\approx {\tilde{\text{A}}}^{k-1}{\text{y}}_{1}=\text{Φ}{\text{Λ}}^{k-1}\text{c} \end{array}$$ (6)

for *k* = 1, 2, …, *N*_*S*_ where **c** is a sequence of weights for which **y**_1_ = **Φc**. Employing the components **Φ**, **Λ**, and **c** from Equation 6 we define a vector function


$$\begin{array}{l} \tilde{\text{x}}\left(t_{k}\right):={\left[{\tilde{x}}_{1}\left(t_{k}\right),\ldots ,{\tilde{x}}_{M}\left(t_{k}\right)\right]}^{T} \end{array}$$

for approximating the term **x**(*t*_*k*_) given by


$$\begin{array}{l} {\tilde{x}}_{i}\left(t_{k}\right):=\overset{R}{\underset{j=1}{\sum }}\lambda_{j}^{k-1}\Phi_{\left(i,j\right)}c_{j},\;k=1,2,\ldots ,N \end{array}$$ (7)

for *i* = 1, 2, …, *M* where λ_*i*_: = **Λ**_(*i, i*)_ and $\text{c}:={\left[c_{1},\ldots ,c_{R}\right]}^{T}$. An approximation $\tilde{X}$ of the original *M*-dimensional time series *X* in Equation 1 is provided by Equation 7 as follows:


$$\begin{array}{l} \tilde{X}:={\left\{\tilde{\text{x}}\left(t_{k}\right)\right\}}_{k=1}^{N} \end{array}$$ (8)

Each $\tilde{\text{x}}\left(t_{k}\right)$ in Equation 8 represents the data point at time *t*_*k*_ reconstructed via DMD, minimizing the influence of noise and encapsulating the quintessential characteristics of the underlying dynamics. For additional details on the computational process, refer to Seo et al. (2020).

#### 2.2.3 DMD components and eigen-frequencies

The signal **x**(*t*_*k*_) is approximated to $\tilde{\text{x}}\left(t_{k}\right)$ given in Equation 7 by applying the DMD algorithm to the following three components: the mode matrix **Φ**∈ℂ^(*MS*) × (*R*)^, the eigenvalue diagonal matrix **Λ**∈ℂ^*R*×*R*^, and the initial amplitude vector **c**∈ℂ^*R*^. Here, **Φ** represents the dominant spatial structure, **Λ**^*k*−1^ represents the temporal evolution, and **c** represents the amplitude of the modes. For convenience, these three components used in the signal approximation are collectively referred to as the “DMD components.” The discrete function **x**(*t*_*k*_):$={\left[x_{1}\left(t_{k}\right),\ldots ,x_{M}\left(t_{k}\right)\right]}^{T}$ in Equation 1, which defines the time series *X*, is extended to a continuous function **x**(*t*) using the DMD components, which is approximated by


$$\begin{array}{l} x_{i}\left(t\right)\approx \overset{R}{\underset{j=1}{\sum }}e^{\left(\log\lambda_{j}\right)t/\Delta t}{\text{Φ}}_{\left(i,j\right)}c_{j},\;t_{1}\leq t\leq t_{n} \end{array}$$

for *i* = 1, 2, …, *M*. Then the *j*^th^ “eigenfrequency”, denoted by ω_*j*_, is given by


$$\begin{array}{l} \omega_{j}:=\frac{\text{Im}\left(\log\lambda_{j}\right)}{2\pi \Delta t},\;j=1,2,\ldots ,R \end{array}$$ (9)

where ω_*j*_ represents the frequency, expressed in cycles per second, of the *j*^th^ mode signal ${\text{Φ}}_{\left(:,j\right)}e^{\omega_{j}t}c_{j}$ corresponding to λ_*j*_, and “Im(·)” denotes the imaginary part of a complex number (Seo et al., 2020).

### 2.3 Features extraction

To ensure sufficient temporal coverage, only participants with total durations longer than 30s were included. This criterion resulted in the exclusion of four subjects (Subject IDs: 15, 64, 65, and 78) with shorter recordings.

Feature extraction proceeds in three steps: (i) epoch construction, (ii) DMD-based windowed segmentation, and (iii) formation of 3D sequenced mode maps.

#### 2.3.1 Creation of epochs

**Figure 1:**
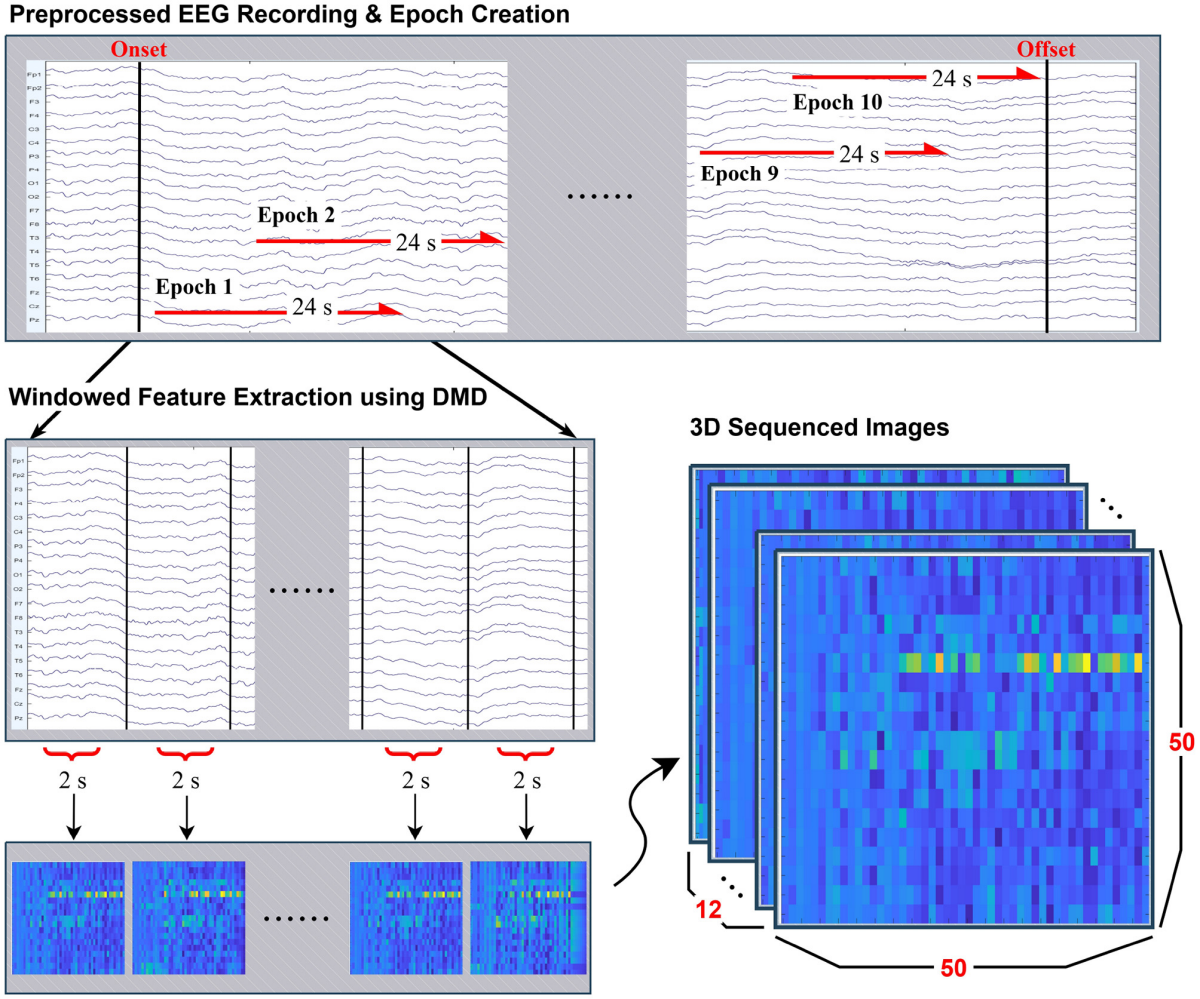
Overview of the preprocessing and feature extraction pipeline. Continuous EEG recordings were segmented into 24-second epochs with overlap. Each epoch was further divided into 2-second windows, from which features were extracted using Dynamic Mode Decomposition (DMD). The resulting spectro-spatial representations were mapped into 50 × 50 grayscale mode maps, and by stacking 12 consecutive segments, each epoch was represented as a 50 × 50 × 12 three-dimensional image for CNN-based classification.

From the stimulus-related EEG recordings described in Table 1, 10 epochs of length 24s were constructed for evert eligible subject by sliding time window across the continuous recordings between photic stimulus onset and offset. Rather than enforcing strictly non-overlapping intervals, a partially overlapping windowing strategy was adopted. This approach ensured that each participant contributed an equal number of epochs while efficiently utilizing the available data, particularly for subjects with limited recording durations. The procedure is illustrated in *Preprocessed EEG Recording & Epoch Creation* in Figure 1.

#### 2.3.2 Segmentation of epochs

Each 24s epoch was further subdivided into 12 segments of length 2s, on which Dynamic Mode Decomposition (DMD) was applied separately to each segment to extract dynamic modes. This process is visualized in *Windowed Feature Extraction using DMD* in Figure 1.

#### 2.3.3 Formation of 3D sequenced images

The resulting mode representations were converted into 50 × 50 grayscale mode maps (resized to 50 × 50 by bilinear interpolation and rescaled to [0, 1]), which were sequentially stacked to form a three-dimensional array of size 50 × 50 × 12 and used directly as numeric tensors for the CNN. When persisted, arrays were stored in a lossless binary format for direct loading into the training pipeline. A visual example of this structured input is provided in *3D Sequenced Images* in Figure 1.

Our uniform epoch selection was designed *a priori* to minimize the direct encoding of stimulus composition as a feature; we then validated this design by summarizing and testing the class–category distribution at the dataset level (see Supplementary Material Section 2; Figure S1), which showed no material imbalance across groups.

*Note that* the 24 s epoch length reflects an empirical trade-off: longer epochs reduced the pool of eligible recordings under the LNSO protocol, whereas shorter epochs degraded classification accuracy; accordingly, 24 s was fixed throughout the analyses as a balance between data retention and performance.

For the image construction step, we performed the DMD decomposition described in Equation 5 on each 2s segment, using a stacking parameter of *S* = 48 and a truncation rank of *R* = 100, which yielded the mode matrix


$$\left[\phi_{1},\phi_{2},\ldots ,\phi_{R}\right]\in {\mathbb{C}}^{M\times R},$$

together with the corresponding eigenfrequencies ω_*j*_ in Equation 9.

Since each mode **ϕ**_*j*_ is uniquely associated with its eigenfrequency ω_*j*_, we sort the set of eigenfrequency–mode pairs {(ω_*j*_, **ϕ**_*j*_)} in ascending order of frequency, and denote the reordered quantities by


$$\left({\tilde{\omega }}_{1},{\tilde{\phi }}_{1}\right),\left({\tilde{\omega }}_{2},{\tilde{\phi }}_{2}\right),\ldots ,\left({\tilde{\omega }}_{R},{\tilde{\phi }}_{R}\right),$$

such that $0\leq {\tilde{\omega }}_{1}\leq {\tilde{\omega }}_{2}\leq \cdots \leq {\tilde{\omega }}_{R}$. Among these, only the pairs satisfying


$$4\;\text{Hz}\leq {\tilde{\omega }}_{j}\leq 40\;\text{Hz}$$

are retained. This selection yields a frequency-filtered mode matrix


$$\Phi^{\left(\text{seg}\right)}=|[{\tilde{\phi }}_{j_{1}},{\tilde{\phi }}_{j_{2}},\ldots ,{\tilde{\phi }}_{j_{K}}]|\in {\mathbb{R}}^{M\times K},$$

where *M* is the number of EEG channels (*M* = 19) and *K* is the number of retained modes. Because *K* varies across segments, the dimensions of **Φ**^(seg)^ are not fixed. To provide a consistent representation, each **Φ**^(seg)^ is interpolated into a 50 × 50 square image, which serves as the basic unit of the CNN input tensor.

Figure 1 illustrates the overall feature extraction pipeline.

It should be noted that the analysis of very low-frequency dynamics is particularly challenging under EO conditions, as alpha suppression and increased variability reduce the stability of spectral estimates in this regime. This challenge is further compounded in our dataset by the heterogeneity of total recording durations across subjects, ranging from approximately 30 seconds to over 150 seconds (Table 1). For subjects with shorter recordings, constructing ten epochs of 24 seconds required substantial overlap, which can impair the reliable estimation of slow rhythms, as overlapping windows may introduce spurious entrainment at the overlap rate (Benjamin et al., 2021). Accordingly, to avoid confounding effects in the delta-band and ensure robust feature extraction, we restricted the DMD-based analysis to the 4–40 Hz range. To provide a fair comparison, however, we also implemented a parallel pipeline using the broader 0.5–40 Hz range, serving as a comparable algorithm. Thus, classification performance was evaluated across both conditions, with the 4–40 Hz range regarded as the main analysis and the 0.5–40 Hz condition used as a benchmark to quantify the contribution of delta-band activity.

This approach is consistent with prior observations that EO EEG is less suited for the study of very low-frequency components, whereas the 4–40 Hz range more reliably reflects attentional and cognitive neural dynamics (Babiloni et al., 2017; Ntetska et al., 2025). Accordingly, our analysis was designed to test this hypothesis by directly comparing classification performance between the 0.5–40 Hz and 4–40 Hz ranges within the same CNN framework.

To better illustrate the characteristics of the frequency-filtered mode matrix **Φ**^(seg)^, we aggregated the results at the class level by averaging the absolute values of the retained DMD modes and their corresponding eigenfrequencies. A side-by-side visual comparison with FFT-based spectra is presented in Section 3, highlighting the differences between the two approaches.

*Note that* all feature-extraction parameters (epoch length, segment duration, number of modes, and image map size) were selected via pilot sweeps/ablation studies under a performance–compute trade-off; settings that increased runtime with only marginal gains were not adopted.

### 2.4 Classification

In this section, the proposed 3D-CNN model architecture is described, the algorithms used for performance comparison are presented, and the validation method and performance metrics are analyzed.

#### 2.4.1 Model architecture

Spectrogram–CNN approaches on long windows primarily learn static spectral–power patterns from a single time–frequency image (Stefanou et al., 2025). In EO with photic stimulation, however, our objective is to track *segment-to-segment changes in inter-sensor coherence*. Since whole-epoch DMD is computationally prohibitive, we compute DMD on short segments and stack the resulting maps; a 3D CNN then acts jointly across the two spatial axes and the segment axis. This induces an explicit modeling bias toward the *temporal evolution of coherence under stimulation*, in contrast to long-window spectrogram models that emphasize static long-epoch power.

The input to the three-dimensional (3D) CNN model consists of 12 sequential 50 × 50 grayscale image with dimensions, 50 × 50 × 12 × 1 (height × width × depth × channel), where the height represents resized brain channels (originally 19), the width represents resized DMD modes between frequency 4–40 Hz, the depth corresponds to the temporal sequence of 12 frames, and the channel corresponds to a single grayscale channel.

The proposed 3D CNN model architecture begins with a 3D image input layer that incorporates z-score normalization. The initial 3D convolutional layer uses 16 filters, each of size 3 × 3 × 3. This is followed by a batch normalization layer to stabilize learning and reduce the internal covariate shift. To introduce non-linearity, a ReLU activation function is applied. This is followed by a MaxPooling layer with a pool size of 2 × 2 × 2 and the same padding to reduce spatial dimensions, maintaining critical features while reducing computation.

The model proceeds with two consequent blocks with 3D convolutional layer, increasing the number of filters to 32 and 64, respectively, while maintaining the 3 × 3 × 3 kernel size. Similarly, batch normalization layers and ReLU activations are used, followed by another MaxPooling layer with a pool size of 2 × 2 × 2 and the same padding.

Following the three convolutional layers, a dropout rate of 0.25 is used to ensure robust feature extraction and avoid overfitting.

Now, the network transitions to the fully connected stage. The extracted features are flattened into a 1D vector and passed to a fully connected layer with 128 units, followed by a ReLU activation function and a dropout rate of 0.20 to improve robustness. The next stage includes a fully connected layer with 64 units, again followed by ReLU activation and a dropout rate of 0.25 to further reduce overfitting.

Finally, the network concludes with a binary classification via Softmax activation on 2 logits. This activation function is suitable for multiclass classification as it converts the logits into probabilities, with each unit corresponding to one of the two target classes.

The final 3D-CNN configuration was determined after a series of targeted ablation studies that varied architectural and training choices (e.g., depth/width, kernel sizes, activation functions, normalization, dropout, learning rate, and batch size). We selected the present design because it offered a practical balance among accuracy, computational cost, and robustness to overfitting.

Training was capped at 75 epochs, a limit chosen after inspecting learning curves: training loss converged while validation performance stabilized well before that point in most runs, and larger caps yielded only marginal gains at higher cost. Thus, 75 epochs served as a conservative ceiling for all reported experiments.

**Figure 2:**
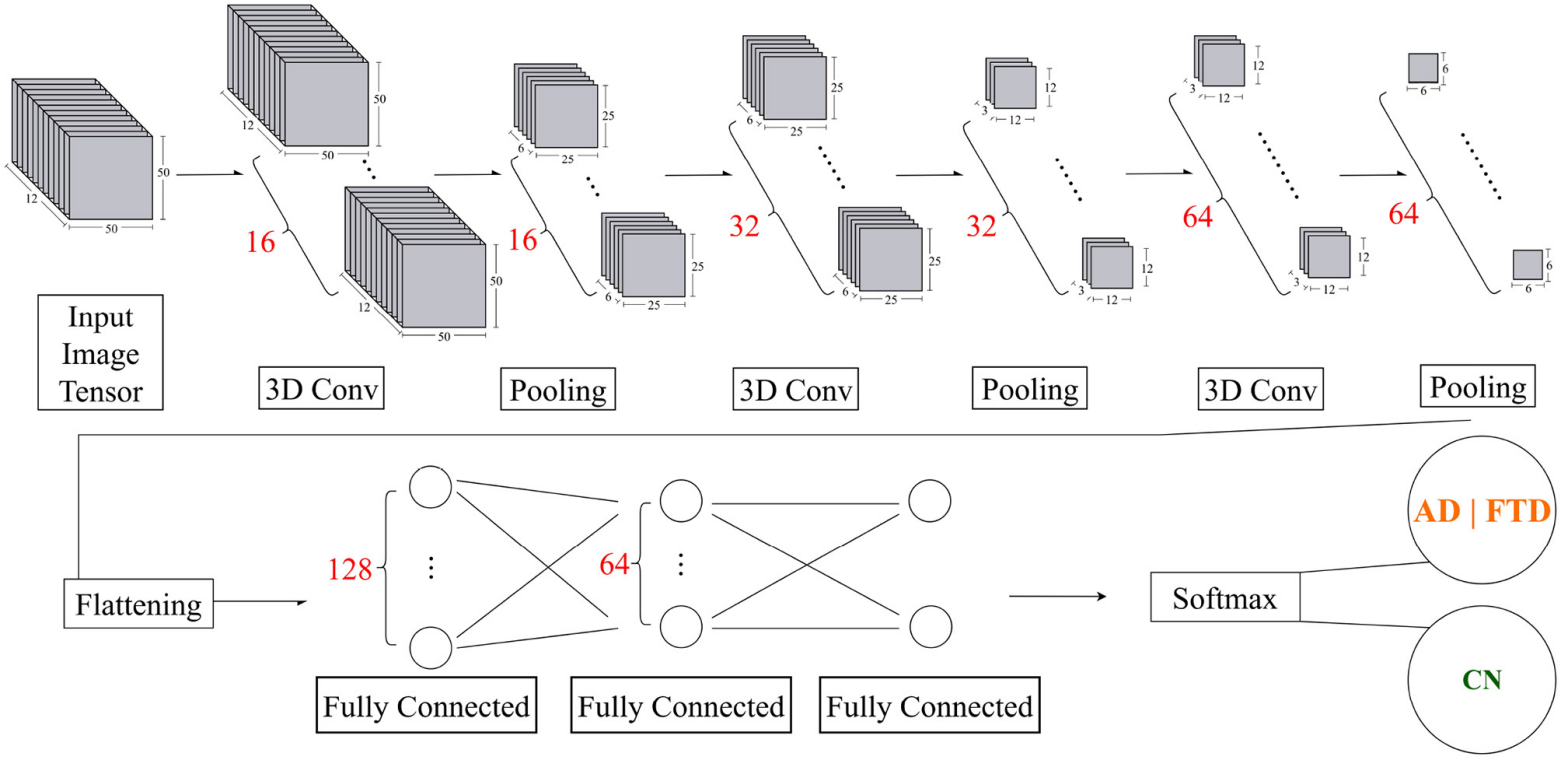
Overview of the proposed 3D CNN model for dementia EEG classification. After the initial input and preprocessing layers, the model comprises three convolutional blocks with progressively increasing filter sizes (16, 32, and 64). Each block is followed by batch normalization, ReLU activation, and 3D max-pooling with a pool size of 2 × 2 × 2. The extracted features are then flattened and passed through fully connected layers with 128 and 64 units, both regularized with dropout (0.20 and 0.25, respectively). A final Softmax activation produces the class probabilities (AD/FTD vs. CN).

The overall architecture of the proposed 3D CNN model is illustrated in Figure 2. The architecture was determined through comprehensive experimentation involving systematic testing of different input dimensions, network configurations, and additional hyperparameter settings.

#### 2.4.2 Comparison algorithms

For comparative analysis, an alternative feature representation was constructed using the conventional Fourier Transform (FFT) in a manner directly parallel to the DMD-based approach. From the stimulus-related EEG recordings described in Table 1, after excluding subjects with total durations shorter than 30s (Subject ID: 15, 64, 65, 78), epochs of 24s were first defined for each subject. Then each epoch was divided into 12 overlapping 4s windows with a 2s step size, producing 12 segments per epoch consistent with the segmentation depth used for DMD.

For each 4s segment (2,000 points at a 500Hz sampling rate), the power spectral density (PSD) was computed separately for each channel using the FFT. The resulting PSD estimates were then converted to decibel units (10log_10_ scale), which is a standard practice in EEG spectral analysis to compress the dynamic range and improve interpretability across frequency bands.

To systematically assess the role of low-frequency components, two input sets were constructed using different retained ranges: 0.5–40 Hz and 4–40 Hz. For each condition, the corresponding spectra were mapped into 50 × 50 × 1 grayscale images, and by stacking 12 consecutive segments, each epoch was represented as a three-dimensional array of size 50 × 50 × 12 × 1. The input sets were then independently evaluated under the same CNN architecture to ensure a fair comparison.

#### 2.4.3 Validation methodology, classification problems, and performance metrics

In EEG classification, conventional *k*-fold cross-validation can cause data leakage, as it splits samples randomly without considering the subject boundaries. This can result in data from the same subject appearing simultaneously in both the training and test folds, leading to artificially inflated performance due to subject-specific features. To avoid this issue, we adopted a leave-*N*-subjects-out (LNSO) validation strategy, which is a generalization of the leave-one-subject-out (LOSO) approach. In LNSO, whenever a subject is selected for validation, all data from that subject's are excluded from the training set. This separation provides a more faithful estimate of cross-subject generalization, which is a central concern in EEG-based studies.

For each binary classification task, the dataset was divided into five equally sized partitions per group, which we refer to collectively as one *batch*. A total of five such batches were constructed. Within each batch, one partition from every group was held out as the testing set, while the remaining four partitions were used for training. This procedure was repeated until each partition had served once as the test set, resulting in five folds per batch. To ensure stability of the training process, model learning was repeated five times for each fold, and the average performance was recorded. Consequently, for a single classification task (e.g., AD/CN), the experimental protocol consisted of 5 × 5 × 5 runs (five batches × five folds per batch × five training repetitions per fold). Considering the three classification tasks (AD/CN, FTD/CN, and (AD+FTD)/CN), the entire study involved a total of 5 × 5 × 5 × 3 experimental runs.

The binary classification scenarios considered were: AD/CN, FTD/CN, and (AD+FTD)/CN. For each case, the following evaluation metrics were reported: accuracy (ACC), precision (PPV), recall (TPR), and F1-score (F1). After completing all runs, the cumulative confusion matrix was constructed, from which these metrics were derived.

It should be noted that in the (AD+FTD)/CN scenario, the number of samples in the combined (AD+FTD) group was nearly twice as large as that of the CN group. Such an imbalance can cause misleading evaluation, since the metrics expressed in percentages may appear balanced even when the minority class is poorly classified. To address this issue, rather than artificially doubling the number of CN epochs, we reduced the number of epochs per AD and FTD subject from 10 to 5, thereby bringing the overall class proportions into balance. This adjustment ensured that performance metrics reflected true classification ability rather than being inflated by class imbalance.

#### 2.4.4 Experimental setup

The experimental protocol and demographic characteristics of the dataset can be found in Section 2.1. All preprocessing, feature extraction, CNN classification, and spectrogram generation were conducted in MATLAB R2025a using the Signal Processing, Statistics and Machine Learning, and Deep Learning toolboxes. Model training was accelerated using the Parallel Computing Toolbox on a NVIDIA RTX 3060 GPU.

## 3 Results

In this section, the performance of the proposed model for each task examined will be reported, along with the performance of the comparison algorithms.

**Figure 3:**
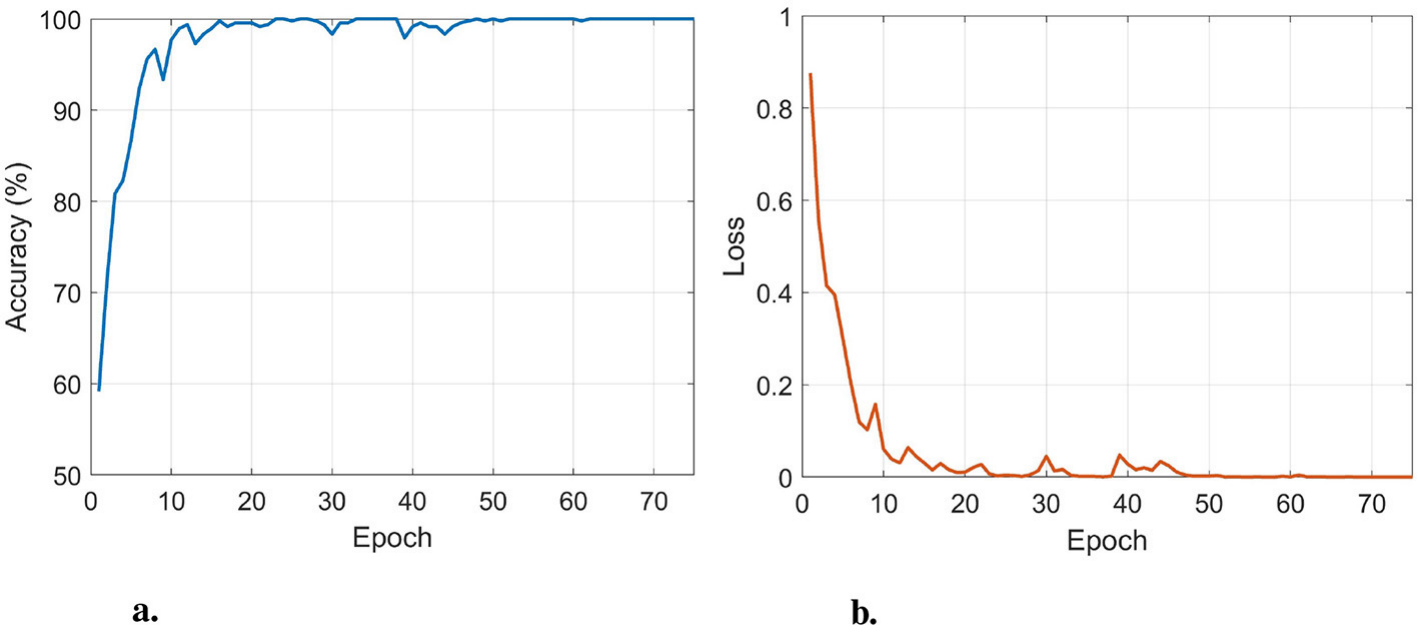
The train accuracy **(a)** and train loss **(b)** in the AD/CN task, with respect to the number of epochs. An optimal performance is obtained at 75 epochs.

To determine the optimal number of training epochs, an 80%–20% train–test split was used for both AD/CN and FTD/CN tasks, and the epoch achieving the highest test accuracy was selected. The optimal value was 75, which was adopted for all leave-*N*-subjects-out (LNSO) runs. The mini-batch size and learning rate were set to 16 and 0.0005, respectively, to minimize training loss while maintaining high generalizability. Figure 3 presents the performance of the classifier with respect to the number of epochs for the AD/CN task.

Table 2 summarizes the accuracy results for each of the five batches for AD/CN, FTD/CN, and (AD+FTD)/CN, including the average accuracy across the batches. A 95% confidence interval is also provided. The average accuracy for the FTD/CN task (77.06%) was higher than that for the AD/CN task (74.23%). For the (AD+FTD)/CN task, the average accuracy was 73.32%.

**Table 2:** Performance in terms of accuracy for the classification tasks: AD/CN, FTD/CN, and (AD+FTD)/CN.

**Test accuracy**	**AD/CN**	**FTD/CN**	**(AD+FTD)/CN**
Batch 1	72.15%	78.16%	70.66%
Batch 2	73.41%	75.12%	74.55%
Batch 3	77.28%	79.71%	73.80%
Batch 4	74.61%	76.32%	74.58%
Batch 5	73.69%	74.01%	73.02%
Average	74.23%	77.06%	73.32%
Std	1.92%	1.61%	1.62%
Margin of Error (MoE) of 95% CI	2.38%	2.00%	2.01%
Lower bound	71.85%	75.06%	71.31%
Upper bound	76.61%	79.06%	75.34%

In terms of variability, the FTD/CN task showed the lowest standard deviation at 1.61%, while the AD/CN experiment exhibited the largest deviation of 1.92%, followed by 1.62% for (AD+FTD)/CN. Based on these standard deviations, the 95% confidence intervals were estimated as 71.85–76.61% for AD/CN, 75.06–79.06% for FTD/CN, and 71.31–75.34% for (AD+FTD)/CN. These results indicate relatively stable performance across all three classification settings, with FTD/CN yielding the highest accuracy on average.

Table 3 shows that the AD/CN task achieved balanced performance across metrics, with an average precision of 73.73%, recall of 73.95%, and F1 score of 73.83%. In comparison, the FTD/CN task exhibited the highest overall performance, achieving an average precision of 76.29%, recall of 77.08%, and F1 score of 76.50%. In contrast, the combined (AD+FTD)/CN task obtained the lowest metrics, with an average precision of 73.27%, recall of 74.46%, and F1 score of 73.13%. These findings indicate that, although all three tasks yielded consistent results, the FTD/CN task was comparatively the most separable under the proposed framework.

**Table 3:** Precision, recall, and F1 score for each class, for the tasks AD/CN, FTD/CN, and (AD+FTD)/CN.

**Metric**	**Group**	**AD/CN**	**FTD/CN**	**(AD+FTD)/CN**
Precision	AD	78.67%	-	-
	CN	68.79%	83.53%	62.77%
	FTD	-	69.05%	-
	AD+FTD	-	-	83.76%
	**Total**	**73.73%**	**76.29%**	**73.27%**
Recall	AD	76.57%	-	-
	CN	71.33%	76.81%	78.55%
	FTD	-	77.35%	-
	AD+FTD	-	-	70.37%
	**Total**	**73.95%**	**77.08%**	**74.46%**
F1 score	AD	77.61%	-	-
	CN	70.04%	80.03%	69.78%
	FTD	-	72.97%	-
	AD+FTD	-	-	76.48%
	**Total**	**73.83%**	**76.50%**	**73.13%**

The final row of each section represents the averaged metric for all the classes of the task.

**Figure 4:**
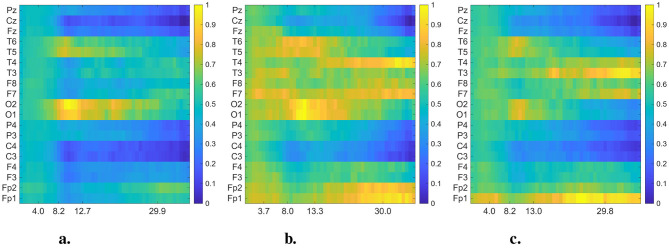
Averaged and normalized DMD mode heatmaps for each group (CN, AD, and FTD) in the 0.5–40 Hz frequency range. The color intensity indicates the magnitude of the DMD modes. In each heatmap, rows correspond to EEG channels, while columns represent the averaged eigenfrequencies associated with the DMD modes. **(a)** Healthy control. **(b)** Alzheimer's disease. **(c)** FTD.

**Figure 5:**
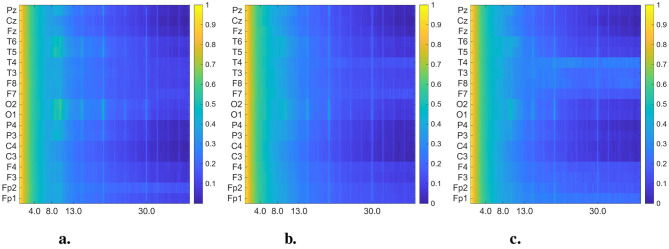
Averaged and normalized FFT heatmaps for each group (CN, AD, and FTD) in the 0.5–45 Hz frequency range. Each row corresponds to an EEG channel, while each column corresponds to a frequency bin, labeled in Hertz (Hz). To improve visual interpretability across frequency bands, the power spectral densities were log-transformed (10 log_10_ scale) before normalized. **(a)** Healthy control. **(b)** Alzheimer's disease. **(c)** FTD.

In Figures 4, 5, we present the averaged DMD- and FFT-based heatmaps for each group (CN, AD, and FTD). Although both representations summarize class-specific spectral content, they emphasize complementary aspects of the data. The FFT heatmaps primarily capture spectral power distributions, highlighting the dominance of low-frequency activity and channel-specific variations. In contrast, the DMD heatmaps provide a more compact representation that captures spatio-temporal coherent modes, producing smoother spatial patterns across channels, and emphasizing dynamic structures that may not be as clearly visible in the FFT view. Together, these complementary perspectives underscore both the spectral differences and the underlying dynamical alterations associated with Alzheimer's disease and frontotemporal dementia. All detailed interpretations of these averaged heatmaps are provided in Section 4.

To compare and validate the performance of the proposed methodology, we benchmarked it against the baseline results reported in Ntetska et al. (2025), using the best-performing models specified for the AD/CN and FTD/CN cases under EO conditions. Conventional FFT-based feature extraction strategies were also included for completeness. In addition, we included DMD-based alternatives with delta-band activity. The results for the AD/CN, FTD/CN, and (AD+FTD)/CN cases are summarized in Table 4, respectively. For fair evaluation, all methods were trained and validated using a leave-n-subject-out (LNSO) protocol, with a fixed random seed to ensure that each batch used the same test set. The proposed DMD+CNN framework consistently outperformed conventional approaches, most notably in the AD/CN scenario, where excluding the delta-band led to the highest and most stable performance.

**Table 4:** Performance comparison of the proposed methodology with baseline classification methods (Ntetska et al., 2025) for AD/CN and FTD/CN tasks, and with alternative feature extraction strategies for AD/CN, FTD/CN, and (AD+FTD)/CN tasks.

**Task**	**Method**	**Accuracy**	**Precision**	**Recall**	**F1**
AD/CN	Baseline (SVM)	62.50%	57.60%	79.60%	66.80%
	FFT with delta-band	70.69%	69.79%	70.28%	69.94%
	FFT without delta-band	70.56%	69.80%	70.12%	69.91%
	DMD with delta-band	72.96%	72.47%	72.62%	72.54%
	**Proposed: DMD w/o delta-band**	**74.23%**	**73.73%**	**73.95%**	**73.83%**
FTD/CN	Baseline (LightGBM)	71.00%	60.50%	68.00%	67.30%
	FFT with delta-band	74.39%	74.21%	73.88%	73.99%
	FFT without delta-band	73.24%	73.00%	72.60%	72.72%
	DMD with delta-band	76.67%	76.54%	76.02%	76.18%
	**Proposed: DMD w/o delta-band**	**77.06%**	**76.29%**	**77.08%**	**76.50%**
(AD+FTD)/CN	FFT with delta-band	67.43%	67.29%	68.55%	66.94%
	FFT without delta-band	69.85%	69.76%	70.70%	69.59%
	DMD with delta-band	73.25%	73.23%	74.14%	73.14%
	**Proposed: DMD w/o delta-band**	**73.32%**	**73.27%**	**74.46%**	**73.13%**

## 4 Discussion

In this study, a novel deep learning framework for the automatic detection of AD and/or FTD from eyes-open (EO) EEG was proposed. We used the publicly released dataset of stimulus-related EO EEG recordings, which had been preprocessed in advance by the dataset providers according to their published pipeline. The methodology consisted of the following: (a) an epoching step in which 24-second segments were defined and further subdivided into twelve overlapping 2-second windows; (b) a feature extraction step based primarily on Dynamic Mode Decomposition (DMD) restricted to the 4–40 Hz range, with conventional FFT-based spectrograms (4-second windows) constructed as comparable baselines under two passbands (0.5–40 Hz and 4–40 Hz); and (c) a convolutional neural network (CNN) trained and evaluated using a subject-wise leave-*N*-subjects-out (LNSO) validation protocol. Side-by-side comparisons within the same CNN architecture indicated that the DMD-derived representations yielded more stable performance for AD under EO conditions while remaining competitive for FTD, suggesting that suppressing the delta-band and modeling spatio-temporal coherent modes mitigates EO-specific challenges and complements conventional spectral-power features.

In our experiments, unlike previous EC-based work using FFT spectrograms, which reported strong performance in AD classification but notably weaker results for FTD (Stefanou et al., 2025), our EO-centered approach demonstrated a different trend. Specifically, the AD/CN task yielded consistent performance with an average accuracy of 74.23% and an F1 score of 73.83%, while the FTD/CN task achieved even higher results (accuracy 77.06%, F1 score 76.50%). This indicates that, under EO conditions, FTD patients may exhibit more discriminable EEG dynamics than AD patients, contrasting with the EC scenario where AD features were more robust. The combined (AD+FTD)/CN classification produced balanced results (accuracy 73.32%, F1 score 73.13%), further underscoring the robustness of the proposed methodology across multiple diagnostic settings.

The comparative results against baseline classifiers and FFT-based approaches are summarized in Table 4. For the AD/CN task, the proposed DMD-based methodology achieved an accuracy of 74.23%, which is markedly higher than the baseline SVM (62.50%) and also exceeded both FFT-based strategies. Notably, in this task, the removal of delta-band (0.5–4 Hz) was crucial: the model without delta components consistently yielded the most reliable performance across precision, recall, and F1 score. This observation supports our initial hypothesis that under EO conditions, delta-band activity is unstable and can obscure the discriminative features relevant for AD detection.

As shown in Section 6.1, a paired-samples *t-test* further confirmed this observation, especially for AD/CN task (ACC with Delta: 72.96%, without Delta: 74.23%). The test revealed a statistically significant improvement in accuracy when delta-band components were excluded [*t*(24) = 2.73, *p* = 0.0117, Cohen's *d* = 0.55]. This result quantitatively supports that, under eyes-open conditions, delta-band activity may introduce noise rather than informative patterns for AD/CN classification.

In the FTD/CN task, our methodology also outperformed the LightGBM baseline, reaching 77.06% accuracy compared to 71.00%. Interestingly, here the effect of delta-band removal was less pronounced, as both DMD and FFT benefited only marginally. This suggests that while FTD dynamics under EO conditions are more distinguishable overall, they are less sensitive to delta contamination than AD-related signals.

For the combined (AD+FTD)/CN setting, both DMD variants produced comparable performance around 73%, with a slight edge for delta exclusion (73.25% vs. 73.32%; Table 4). This contrasts with the subtype-specific analyses, where excluding delta benefited AD/CN and including delta benefited FTD/CN, and suggests that, when AD and FTD are merged, their divergent delta-band signatures may partially offset each other. As a result, delta inclusion captures shared slowing features that help the combined dementia class versus CN, while the overall accuracy remains lower than in subtype-specific models.

Taken together, these findings emphasize that (a) the proposed DMD-based representation offers consistent advantages over conventional FFT-based features and baseline classifiers, and (b) delta-band activity behaves chiefly as a nuisance: its perturbation is stronger in AD.

Additionally, in *exploratory analyses* (Appendix Tables 7, 8), AD/FTD showed a nonsignificant but favorable trend with delta exclusion (mean Δ≈+1.3 pp; Appendix Table 7). By contrast, in the three-class AD/FTD/CN task, performance was *slightly lower when delta was excluded* (overall Δ≈−0.2 pp; Appendix Table 8), reflecting a small, nonsignificant advantage for FTD/CN when delta was retained.

Previous EEG studies using FFT-based spectrograms have consistently reported that Alzheimer's disease is characterized by abnormal alterations in alpha and theta activity, particularly reflected in changes in alpha rhythm, alpha–theta ratios, and, in some cases, beta–theta interactions (Schmidt et al., 2013; Özbek et al., 2021; Fonseca et al., 2011). Building on these established findings, the present study visually examined whether such abnormalities also manifest under EO conditions, using DMD- and FFT-based spectrograms.

From a global spectral perspective, CN participants under EO conditions exhibited the expected suppression of alpha activity accompanied by relatively stronger beta power, in contrast to the dominant posterior alpha peak typically observed in EC (Stefanou et al., 2025). AD patients displayed the generalized slowing pattern that has been consistently reported in EC studies, characterized by increased theta and reduced alpha activity, but in EO the low-frequency components appeared less stable and showed higher variability across epochs. FTD patients also showed persistent theta enhancement, yet with a more pronounced anterior–temporal emphasis and reduced posterior dominance compared to EC. These global differences highlight that EO reduces the reliability of very low-frequency components, while shifting the relative contribution of higher frequency bands.

At the channel level, further distinctions were observed. In CN, alpha power was dominant in the occipital (O1, O2) and posterior temporal (T5, T6) regions under EC, whereas in EO the occipital alpha was suppressed and beta activity became more diffuse, with a partial loss of hemispheric symmetry. In AD, the classical EC pattern of posterior alpha loss and theta increase (O1/O2, T5/T6) was replicated, but under EO, theta power was less consistently localized and beta activity redistributed in a patch-like manner over the frontotemporal areas (F7/F8, T3/T4). In FTD, the EC pattern of frontal–temporal theta elevation and posterior alpha reduction was preserved, but EO recordings revealed relatively stronger anterior–temporal power in the alpha/beta range, together with a further decrease in posterior activity. These channel-specific differences indicate that EO accentuates frontal–temporal abnormalities while diminishing posterior features, thereby altering the discriminability between AD and FTD.

Therefore, both EC and EO conditions reveal a generalized slowing of activity in dementia patients, but EO recordings additionally exhibit reduced posterior alpha hemispheric symmetry and a redistribution of spectral power toward frontal and temporal regions. These alterations highlight the greater complexity and variability of EO dynamics compared to EC, underscoring the need for advanced analytic approaches such as DMD to capture these non-stationary features.

Upon closer inspection of Figure 4, notable distinctions emerge above 13 Hz (beta band) between dementia groups and controls. While CN maintains relatively uniform activity in posterior channels, both AD and FTD display distinct enhancements at “frontotemporal sites,” particularly around “F7/F8” and “T3/T4.” These regions are known to contribute to visual attention and cognitive integration, and previous research supports this functional role. For example, beta-band power over occipital regions correlates with visual attention in elderly participants (Gola et al., 2013). Moreover, altered alpha- and beta-band functional connectivity has been observed in visuospatial memory tasks in MCI and early AD (Fodor et al., 2021). Thus, the channel-specific deviations we observe in the beta range likely reflect differential recruitment of attention-related networks during visual stimulation in dementia.

In addition to the well-established slowing in the theta–alpha range reported in dementia, our DMD-based analysis of EO EEG highlighted distinct high-frequency (≥30 Hz) dynamics. Specifically, correlated activity in the frontal electrodes (Fp1–Fp2) and asymmetric gamma-band patterns in temporal regions (T3/T4, F7/F8) emerged as potential differentiating features between AD and FTD. These alterations were not evident in conventional FFT spectrograms (Figure 5), underscoring the advantage of DMD in capturing spatio-temporal coherent modes beyond static power spectra. Previous studies have suggested that gamma-band activity is closely linked to cognitive and attentional processes and may be disrupted in Alzheimer's disease (Basar, 2013; Adaikkan and Tsai, 2020), while asymmetrical frontal–temporal alterations have also been observed in FTD cohorts (Nishida et al., 2011; Stam, 2005). Our findings suggest that EO EEG, when analyzed with spatio-temporal methods such as DMD, may provide novel gamma-band biomarkers that complement the traditionally emphasized low-frequency alterations.

The novelty of this study lies in the application of Dynamic Mode Decomposition (DMD) to eyes-open (EO) EEG analysis and the use of finely segmented data to construct three-dimensional (3D) input representations. Unlike previous CNN-based work that employed relatively long (30 s) epochs, our approach decomposed the data into shorter segments, thereby enabling DMD to capture spatio-temporal coherent modes while preserving a richer set of spectral–spatial dynamics. This design allowed for the detection of frequency- and channel-specific alterations, including higher-frequency (above 30 Hz) activity and strong inter-channel correlations, particularly between frontal sites (Fp1–Fp2), that were not as apparent in FFT-derived spectrograms.

An important finding of this study concerns the role of the delta-band. Specifically, removing delta activity (0.5–4 Hz) led to a clear improvement in AD classification performance, whereas FTD classification showed little change or even modest gains when delta was retained. This pattern is consistent with low-frequency ( ≤ 4 Hz) interference exerting a comparatively stronger effect in AD under eyes-open visual stimulation (>5 Hz), while its influence in CN/FTD appears limited.

When compared to prior work, a contrasting trend emerges. In eyes-closed (EC) conditions, CNN-based spectrogram approaches achieved relatively high accuracies for AD (accuracy = 79.45%, F1 score = 77.60%) but lower values for FTD (accuracy = 72.85%, F1 score = 67.85%), with the delta-band excluded from the spectral representation. In contrast, under EO conditions, baseline FFT studies generally included delta activity, yet showed markedly reduced performance for AD (~60%) while maintaining comparatively higher results for FTD (~71%).

Our DMD-based framework substantially improved these outcomes, mitigating the limitations of small and imbalanced datasets through its dynamic feature extraction and 3D representation. Beyond this overall improvement, our findings highlight a notable condition- and group-specific sensitivity to the delta-band. Specifically, classification for FTD benefitted from the inclusion of delta activity under both EC and EO conditions, whereas AD classification improved when delta was excluded in EO. Although we do not attribute these effects solely to delta activity, this divergence represents a remarkable observation that underscores the need for careful consideration of spectral band selection in dementia-related EEG analysis.

Nevertheless, residual constraints remain, including variability in recording durations across subjects (ranging from approximately 30 to 150 seconds; see Table 1), which restricted the attainable performance ceiling. These limitations emphasize the importance of standardized, longer-duration EO datasets for future research.

In summary, DMD provides distinct advantages over FFT by uncovering high-frequency activity and subtle channel-level asymmetries that are not easily observed with conventional approaches. Furthermore, the differential influence of the delta-band on AD/FTD classification highlights its potential role as a pathophysiological marker. Taken together, these findings demonstrate that EO EEG, when analyzed with advanced decomposition methods such as DMD, offers a promising avenue for the development of robust and physiologically grounded biomarkers for dementia classification.

In prior CNN-based work (Stefanou et al., 2025), it was acknowledged that alternative time–frequency transforms, such as the wavelet transform, could potentially improve time–frequency resolution while addressing temporal inconsistencies in EEG recordings. In this study, we extended this perspective by employing DMD, which inherently provides both spectral information and spatio-temporal coherence. This dual capacity allowed us to better capture the complex dynamics of EO EEG, and we confirmed that DMD-derived features yielded competitive classification performance compared to conventional FFT-based spectrograms.

Furthermore, while EEG coherence and other connectivity measures have been proposed as useful features for dementia classification, DMD partially alleviates the need for explicit connectivity modeling by simultaneously analyzing all channels and extracting coherent spatio-temporal modes. This property offers an indirect yet effective way of incorporating connectivity-like information without additional preprocessing steps.

However, DMD also presents important limitations. The method is computationally more demanding than FFT, both in terms of processing time and memory requirements. Although the use of shorter segmentation windows (2 s) was found to provide the best trade-off between stability and efficiency, DMD at longer windows (e.g., 4 s) achieved slightly better performance but at the cost of substantially slower computation and increased memory load. These constraints may limit the scalability of DMD for large datasets or real-time applications. Future work should therefore focus on developing optimized or approximate implementations of DMD to improve computational feasibility while retaining its unique ability to capture dynamic EEG features.

Future research will aim to expand the dataset by combining the existing EO recordings with the previously released EC data, thereby enabling a more comprehensive exploration of dementia-related EEG dynamics. In addition, a cross-condition transfer learning strategy will be investigated, in which pre-trained layers from both EC- and EO-based models are reused and fused, followed by the addition of task-specific layers for joint fine-tuning. This approach harnesses complementary representations learned under different conditions and is expected to improve the robustness and generalization of the final classifier. From a methodological perspective, the time efficiency of DMD will be revisited. A hybrid strategy may be considered, in which longer windows are analyzed using FFT for stable spectral estimation, whereas shorter windows are processed with DMD to extract fine-grained dynamic features. Finally, future studies will extend the current methodology to other forms of dementia, thereby examining whether disease-specific oscillatory patterns and spatial signatures can be consistently identified across conditions.

## 5 Conclusion

This study investigated eyes-open (EO) EEG recordings for the classification of Alzheimer's disease (AD) and frontotemporal dementia (FTD), a condition that has traditionally been considered more challenging than eyes-closed (EC) EEG. While previous FFT-based approaches on the same dataset reported limited performance for AD under EO conditions, our work introduced a CNN-based framework with Dynamic Mode Decomposition (DMD)-derived features.

By segmenting EEG into short windows and transforming them into long-epoch DMD representations, we enabled CNNs to capture spatio-temporal dynamics beyond conventional spectral power. In addition, we systematically examined the role of low-frequency activity and found that excluding the delta-band improved AD/CN classification accuracy, raising performance above previously reported FFT-based baselines. These results suggest that both delta suppression and DMD-based feature representations are crucial for enhancing EO EEG discriminability.

Our findings suggest that EO EEG, when modeled with appropriate feature extraction and learning strategies, can provide reliable classification performance for AD, approaching levels previously observed for FTD.

## Data Availability

Publicly available datasets were analyzed in this study. This data can be found here: https://openneuro.org/datasets/ds006036/versions/1.0.5.
